# Leaf Size Development Differences and Comparative Transcriptome Analyses of Two Poplar Genotypes

**DOI:** 10.3390/genes12111775

**Published:** 2021-11-09

**Authors:** Lei Zhang, Jiujun Du, Xiaolan Ge, Demei Cao, Jianjun Hu

**Affiliations:** 1 State Key Laboratory of Tree Genetics and Breeding, Key Laboratory of Tree Breeding and Cultivation of National Forestry and Grassland Administration, Research Institute of Forestry, Chinese Academy of Forestry, Beijing 100091, China; zhang_lei1142@163.com (L.Z.); dujiujun0517@163.com (J.D.); gedalan@126.com (X.G.); caodm_ygm@163.com (D.C.); 2 Collaborative Innovation Center of Sustainable Forestry in Southern China, Nanjing Forestry University, Nanjing 210037, China

**Keywords:** *Populus*, leaf size, cell cycle, cell division, growth-regulating factor

## Abstract

The plant leaf, the main organ of photosynthesis, is an important regulator of growth. To explore the difference between leaf size of *Populus* *deltoides* ‘Danhong’ (Pd) and *Populus simonii* ‘Tongliao1’ (Ps), we investigated the leaf length, leaf width, leaf thickness, leaf area, leaf mass per area (LMA), and cell size of leaves from two genotypes and profiled the transcriptome-wide gene expression patterns through RNA sequencing. Our results show that the leaf area of Pd was significantly larger than that of Ps, but the epidermal cell area was significantly smaller than that of Ps. The difference of leaf size was caused by cell numbers. Transcriptome analysis also revealed that genes related to chromosome replication and DNA repair were highly expressed in Pd, while genes such as the *EXPANSIN* (*EXPA*) family which promoted cell expansion were highly expressed in Ps. Further, we revealed that the growth-regulating factors (*GRFs*) played a key role in the difference of leaf size between two genotypes through regulation of cell proliferation. These data provide a valuable resource for understanding the leaf development of the *Populus* genus.

## 1. Introduction

The leaf is an important organ for plant photosynthesis, respiration and transpiration. Its size and shape will affect photosynthetic efficiency and plant growth closely related to plant growth potential, nutrient supply, yield, quality and resistance [1]. Leaves originate from the leaf primordia. The morphogenesis of leaf primordia can be divided into three stages: initial stage, establishment of leaf polarity, and cell expansion [2,3].

Cell size and cell number are the determinants of leaf size, and the process of cell expansion and differentiation is regulated by plant hormones, growth-regulating factors (*GRFs*), *TEOSINTE BRANCHED1*/*CYCLOIDEA*/*PCF* (*TCP*), *WUSCHEL RELATED HOMEOBOX* (*WOX*) and other regulatory factors [3,4]. *GRFs* regulate cell proliferation by means of redundancy to promote leaf growth and are regulated by miR396. Leaves of a *grf1*/*2*/*3* triple mutant are much smaller than those of wild type, whereas overexpression of *AtGRF1*, *AtGRF2*, *AtGRF3* or *AtGRF5* results in larger leaves in *Arabidopsis* [5,6]. Overexpression of the *ZmGRF10* leads to reduction in leaf size and plant height [7]. *CsWOX1* controls auxin transport in leaf veins through *CsPID1*, and interacts with *CsTCP4a* to cause abnormal cucumber leaf development [8]. Structural genes participated in leaf development. For example, the D-type cyclin *CYCD3* plays an important role in determining cell number in developing lateral plant organs by controlling the G1/S transition, and contributes to the alternative event of cell production to cell expansion for stable tissue growth [9].

Poplar is an important economic, ecological and energetically-relevant tree species widely distributed across the world [10]. Environmental differences can allow plants to adapt continuously to parameter genotype and phenotypic variation [11]. *P*. *deltoides* ‘Danhong’ is a fast-growing insect-resistant poplar with large, thin and deltoid leaves that are held by a long and flat petiole [12,13]. *P*. *simonii* is a native tree species in northern China, which has good cold and better drought tolerance with small, thick, and ovate leaves that are attached to a short and stout petiole [14]. Although Sun et al. [13] identified 109 quantitative trait locus (QTLs) on 18 linkage groups for leaf morphological traits of the *P*. *deltoides* ‘Danhong’ × *P*. *simonii* ‘Tongliao1’ pedigree, the most likely key regulatory genes have not been screened.

In order to analyze the reasons for the significant difference in leaf size between *P*. *deltoides* ‘Danhong’ and *P*. *simonii* ‘Tongliao1’, and to explore the important regulatory genes that may be involved in leaf development, we completed the leaf phenotypic investigation and performed comparative transcriptome analysis. We identified differentially expressed genes and analyzed transcription factors (TFs) involved in leaf development. The results of this investigation may provide further basis to elucidate leaf development in cell proliferation and expansion of *P*. *deltoides* ‘Danhong’ and *P*. *simonyi* ‘Tongliao1’.

## 2. Materials and Methods

### 2.1. Plant Material

All experiments were conducted in two poplar species (*P*. *deltoides* ‘Danhong’ (Pd) and *P. simonii* ‘Tongliao1’ (Ps), Supplementary Figure S1). The 15 cm long cuttings were prepared from 1-year-old twigs of the two species and then vegetatively propagated and planted (0.5 m × 1 m in a botanical garden) in the Chinese Academy of Forestry, Beijing, China (116°44′17.24″ E, 39°43′34.70″ N). On 20 May 2019, the unexpanded leaves at the top of the 3-month-old saplings (about 60 cm) were immediately collected in liquid nitrogen for RNA sequencing (RNA-Seq) and then kept at −80 °C until use. A total of 6 samples (2 genotypes × 3 biological replicates) were collected.

### 2.2. Microscopic Analysis

The mature leaves of Pd and Ps were used for microscopic analysis. For each genotype (Pd and Ps), 3 biological replicates were selected for phenotypic statistics, and 3 leaves were selected for each plant. To determine epidermal cell size, the collected leaves in the center of the leaf blade, between the midvein and leaf margin, were fixed with FAA (acetic acid: 96% alcohol; 1:6) and cleared with chloral solution (200 g chloral hydrate, 20 g glycerol and 50 mL dH_2_O), as previously described by Horiguchi et al. [15]. In order to observe the size of leaf epidermal cells, we selected 15 to 20 fields (239.41 μm × 360 μm) for each site to calculate the number of cells. The epidermal cell size was equal to the leaf area/the epidermal cell number (239.41 μm × 360 μm/number of cells). The base of the leaf blade was embedded in 5% agarose for microscopic observation. Leaf cross sections with 40 μm were obtained using a rotary microtome (Leica VT1200S, Wetzlar, Germany) and the sections were stained for 2 min in 0.05% Toluidine Blue O. All the sections were examined with a microscope (Olympus BX51, Tokyo, Japan). Leaf size, thickness and palisade cell thickness were calculated using ImageJ software [16]. Leaf mass per area was equal to the leaf dry weight divided by the total leaf area (mg/cm^2^).

### 2.3. RNA-Seq and Identification of Differentially Expressed Genes

Total RNA of 6 samples (Pd1, Pd2, Pd3, Ps1, Ps2, and Ps3) was isolated using the RNAprep Pure Plant Plus Kit (TIANGEN, Beijing, China). A total amount of 3 µg RNA per sample was used as input material for the RNA sample preparations. Sequencing libraries were generated using NEBNext^®^ Ultra™ RNA Library Prep Kit for Illumina^®^ (NEB, Ipswich, MA, USA). The library preparations were sequenced on an Illumina Hiseq platform (LC Sciences, Houston, TX, USA) and 125 bp/150 bp paired-end reads were generated. The clean reads were mapped to the reference genome *P*. *trichocarpa* v3.0 using TopHat v2.0.12 [17]. Sequencing datas were available in the NCBI SRA database (SRA number: SRR13959619 to SRR13959624). The differentially expressed genes were selected if they were |log2(fold change)| ≥ 1 and (*p*-value ≤ 0.05) by the R package DEGSeq [18]. Gene Ontology (GO) enrichment analysis of DEGs was implemented by the GOseq R package [19]. We used KOBAS software to test the statistical enrichment of DEGs in KEGG pathways [20]. Corrected *p*-value < 0.05 was considered indicative of GO and KEGG enrichment analysis in DEGs.

### 2.4. Evaluation of Gene Expression Using qRT-PCR

To verify the accuracy of transcriptome data, we performed qRT-PCR. qRT-PCR was performed on the Roche LightCycler 480 (Roche Applied Science, Penzberg, Germany). The first stand cDNA was acquired using FastKing gDNA Dispelling RT SuperMix (Tiangen, Beijing, China) with 1 µg mRNA. The specific primers of leaf size-related genes used for qRT-PCR were designed using Primer 3.0 (Table S1). *PtrActin* (Potri.001G309500) was used as the internal control [14]. The qRT-PCR system consisted of SYBR^®^ Premix Premix Ex Taq^TM^II qPCR master mix (TaKaRa, Dalian, China). The reaction procedure was: 95 °C for 30 s, 40 cycles of 95 °C for 5 s, 60 °C for 20 s, and 72 °C for 20 s. The data were analyzed by the 2^−ΔΔCT^ method [21].

### 2.5. Statistical Analyses

The significant differences among treatments of phenotypic data were evaluated at *p* = 0.05 by Duncan’s multiple range test, performed by using SPSS (PASW, Windows version 18.0) after passing the homogeneity test. Histograms and line graphs were performed using Microsoft Excel 2010 software.

### 2.6. Evolution and Expression Analysis of GRFs

Differential expression *PtrGRFs* were used for expression and evolutionary analysis. The tissue expression data of different *PtrGRFs* in this study were obtained from Phytozome(https://phytozome.jgi.doe.gov/pz/portal.html, accessed on 11 November 2020). The evolutionary tree was constructed by MEGA 6.0 with the neighbor joining method (NJ) of *Arabidopsis thaliana*, *P*. *trichocarpa* and *Salix purpurea* [22]. Homologous gene sequences were obtained by Blast in Phytozome. The protein–protein interaction (PPI) data were obtained from the STRING database (https://string-db.org/, accessed on 15 December 2020) [23]. Cytoscape was used to visualize the resulting networks [24]. The gene IDs of *PtrGRFs* used in this study were as follows: *PtrGRF1*/*2Ac* (Potri.007G007100), *PtrGRF1*/*2b* (Potri.014G007200), *PtrGRF1*/*2c* (Potri.002G115100), *PtrGRF2* (Potri.002G115300), *PtrGRF6a* (Potri.006G143200), *PtrGRF6b* (Potri.018G065400), *PtrGRF8* (Potri.001G082700), *PtrGRF11b* (Potri.019G042300), *PtrGRF12a* (Potri.001G114000), *PtrGRF12b* (Potri.003G118100).

## 3. Results

### 3.1. The Leaf Phenotype of Pd and Ps

**Figure 1:**
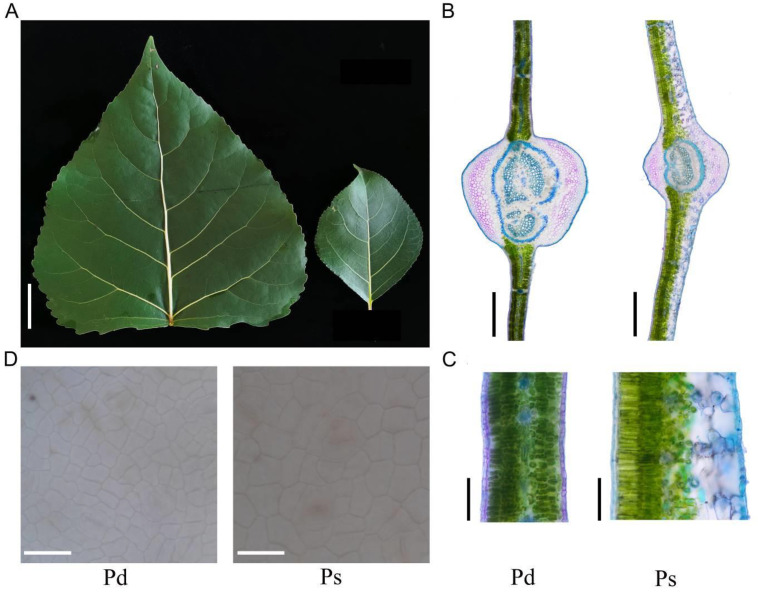
Leaf characteristics of *P*. *deltoides* ‘Danhong’ and *P. simonii* ‘Tongliao1’. (**A**) Morphology of mature leaves. (**B**,**C**) The anatomical structure of leaf cross section. (**D**) Photograph of epidermal cells. Bar = 3 cm (**A**), 500 μm (**B**), 100 μm (**C**), and 50 μm (**D**). Pd, *P*. *deltoides* ‘Danhong’; Ps, *P. simonii* ‘Tongliao1’.

The morphological measurement of growth and leaf morphological characters of Pd and Ps were significantly different. The leaf length, leaf width, and leaf area of Pd were 19.53 cm, 18.45 cm, and 253.22 cm^2^, respectively, which were significantly larger than Ps (Figure 1A, Table 1). The fresh weight of Pd was about 5 times of that of Ps, but its leaf mass per area was 0.67 mg/cm^2^, which was less than Ps (Table 1). Anatomical structure showed that the Pd had two layers of palisade tissues, the lower layer of palisade tissue was sparsely arranged, while Ps only had upper palisade tissue but more tightly arranged (Figure 1B,C). Although the leaf area of Pd was large, the area of epidermal cell was only 399.90 μm^2^, which was significantly less than 753.16 μm^2^ (Figure 1D, Table 1).

### 3.2. Defining Differentially Expressed Genes and Functional Annotation

To further elucidate the molecular basis of leaf shape between Pd and Ps, a global transcriptomic analysis was performed using RNA-Seq. A total 329, 434, 054 clean reads (49. 42 Gb) were obtained from six samples. The GC content of Pd and Ps was 43.87% and 44.25%, and the Q30 was 94.77% and 94.55%, respectively (Supplementary Table S2).

**Figure 2:**
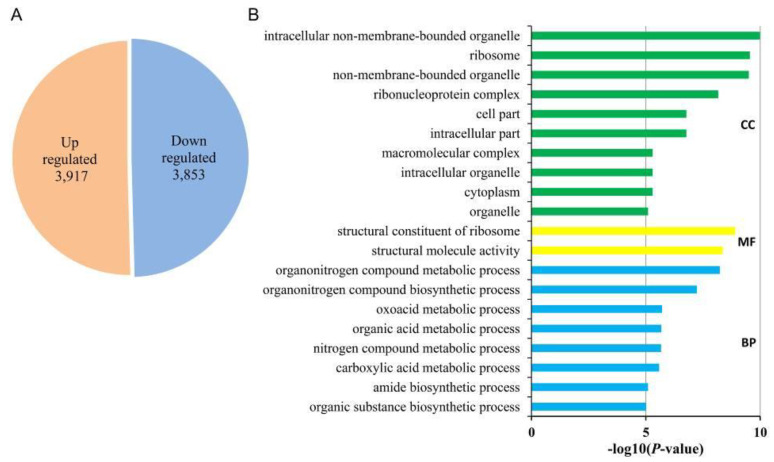
The differentially expressed genes (DEGs) in leaves of *P*. *deltoides* ‘Danhong’ and *P. simonii* ‘Tongliao1’. (**A**) Statistics of DEGs in leaves. (**B**) Enriched gene ontology (GO) terms of DEGs in leaves. BP: biological process, CC: cellular component, MF: molecular function.

A total 7700 differentially expressed genes (DEGs) were identified in Pd vs. Ps (|log2FoldChange| ≥ 1 and *p*-value < 0.05), including 3917 up- and 3853 down-regulated genes (Figure 2A). The DEGs were annotated with GO terms to predict the function, including biological processes (BP), molecular functions (MF) and cellular components (CC) (Figure 2B). In the BP part, subcategories of “organonitrogen compound metabolic and biosynthetic process” (GO:1901566 and GO:1901564), “oxoacid metabolic process” (GO:0043436), and “organic acid metabolic process” (GO:0006082) were significantly enriched. The “structural constituent of ribosome” (GO:0003735) and “structural molecule activity” (GO:0005198) were enriched in the MF part. In the CC category, “intracellular non-membrane-bounded organelle” (GO:0043232), “ribosome” (GO:0005840) and “ribonucleoprotein complex” (GO:0030529) were considerably enriched (Supplementary Table S3).

**Figure 3:**
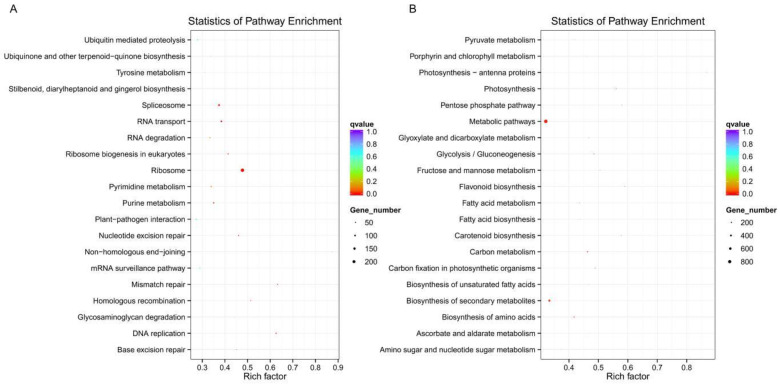
KEGG pathway enrichment analysis of two genotypes. (**A**) Pd vs. Ps up-regulation DEGs enriched KEGG pathway scatterplot. (**B**) Pd vs. Ps down-regulation DEGs enriched KEGG pathway scatterplot. Rich factor refers to the ratio of the number of DEGs located in the KEGG to all genes in the KEGG pathway.

To further understand the biological functions of the DEGs, we performed pathway analysis based on the KEGG database (Figure 3 and Supplementary Table S4). Similar to GO enrichment, the up-regulation genes in Pd vs. Ps were significantly enriched in “Ribosome” (pop03010), “DNA replication” (pop03030), “Mismatch repair” (pop03430), “Spliceosome” (pop03040), and “RNA transport” (pop03013), which are highly related to cell division and chromosome replication, including the 60S ribosomal protein L15 (*RPL15*, Potri.013G106800) (Figure 3A, Table 2). While the down-regulated genes participated in “Carbon metabolism” (pop01200), “Photosynthesis” (pop00195), “Metabolic pathways” (pop01100), and “Glycolysis/Gluconeogenesis” (pop00010) pathways (Figure 3B). The previous metabolomics showed that *P*. *simonii* enhanced its drought resistance by enhancing non-enzymatic antioxidants, coordinating the cellular carbon/nitrogen balance and regulating wax biosynthesis [25,26]. The up-regulation genes in Ps were widely involved in carbon metabolism and flavonoid biosynthesis. Flavonoids are important secondary metabolites, which may be the inherent reason for Ps with resistance to abiotic stress.

To further analyze the regulatory genes that may be involved in leaf formation, we combined the candidate genes of leaf morphological and physiological traits identified by the high-density QTL mapping of *P. deltoides* ‘Danhong’ × *P*. *simonii* ‘Tongliao1’ in the previous study [13]. The 21 QTLs and 38 DEGs shared with the transcriptome related to leaf length, leaf width, and leaf circumference were found (Supplementary Table S5). For example, leaf width-related Potri.014G145100 (*Chalcone Synthase*, *CHS1*) in qLW-LG14-17, and Potri.015G112200 (*cyclin p4;1*) and Potri.015G112600 (Root hair defective 3 GTP-binding protein, *RHD3*) in qLW-LG15-13 were highly expressed in Ps (Supplementary Table S5).

### 3.3. DEGs Related to Auxin and Gibberellin Regulation of Leaf Development

Auxin and gibberellin are the principal regulators of leaf development. We found 31 DEGs in our datasets that were associated with the auxin and gibberellin pathways, and potentially related to leaf development. Two *YUCCAs*, eleven *AUX*/*IAAs*, two *AUXIN RESPONSE FACTORs* (*ARFs*), and five auxin transport proteins (*PINs*), were related to auxin biosynthesis and the signal pathway. In addition, 14 gibberellin oxidases related to gibberellin biosynthesis were found (Supplementary Table S6). These results indicate that auxin and gibberellin may affect the expression of cellular structure genes or TFs to regulate the leaf development.

### 3.4. The Regulation of Transcription Factors for Cell Cycle

The plant cell cycle is controlled by the activity of complexes consisting of a cyclin-dependent kinase as the catalytic subunit and a cyclin as the regulatory subunit. A-type cyclin dependent kinase (*CDKA*) and D-type cyclin (*CYCD*) are central to the G1/S phase transition in which the cell activates DNA duplication. Twenty-two differentially expressed cyclins were identified between Pd and Ps, including six *CYCA*s, eight *CYCB*s, and eight *CYCD*s. Most of them are highly expressed in Pd, except *CYCD1;1*, *CYCD2;1*, and *CYCD3;1* (Supplementary Figure S2, Table S7). We also successfully identified differential expression of *CYCD4;1* (Potri.015G112200), a candidate gene of QTL leaf size in the previous study, in the two genotypes (Supplementary Table S5).

Cell expansion is an indispensable step determining the final leaf size, which is controlled by different mechanisms at each stage of cell development. Expansin (*EXPAs*), the marker of cell size, was highly expressed in Ps (Supplementary Table S8). The cell wall provides the skeleton for cell support, affects the cell size, and thus determines the leaf size. Several genes related to cell wall biosynthesis were also found, such as cellulose synthase A9 (*CesA9*), LACCASE17 (*LAC17*), and XYLOGLUCAN ENDOTRANSGLUCOSYLASE/HYDROLASE (*XTH*) involved in cellulose, lignin and hemicellulose biosynthesis (Supplementary Table S8).

**Figure 4:**
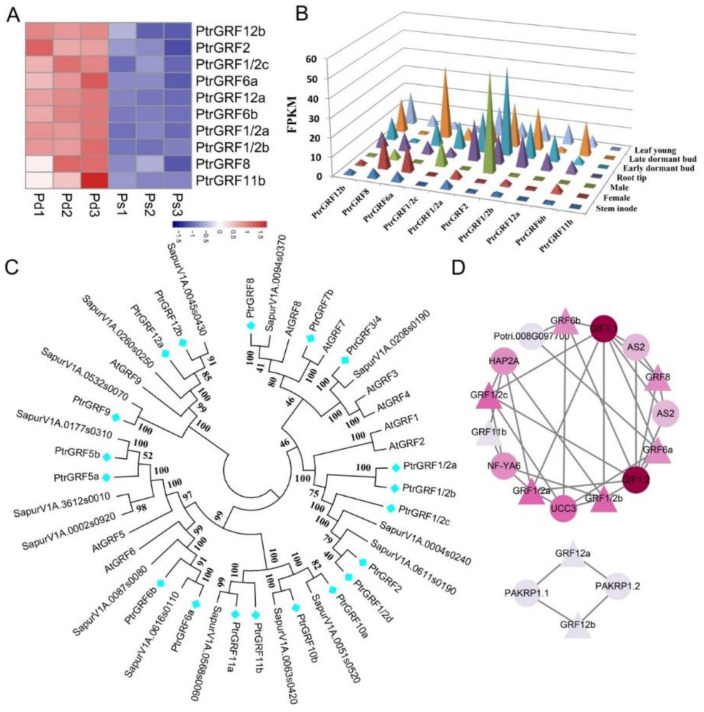
Phylogenetic analysis and expression patterns of the *PtrGRF* family. (**A**) The DEGs of the *PtrGRF* family in Pd and Ps. (**B**) Expression patterns of *PtrGRFs* across various tissues. (**C**) Phylogenetic analysis. The phylogenetic tree was constructed using the full-length amino acid sequences by the neighbor-joining (NJ) method with 1000 bootstrap replicates. (**D**) Protein–protein interaction (PPI) network of differentially expressed *GRFs*. The color from light to dark represents the degree of connection.

Transcription factors are important regulators of plant life activities. In this study, *GRF*, *TCP*, scarecrow (*SCR*) and other transcription factors related to cell cycle were identified, which regulated the expression of the cyclin family. It was found that *PtrGRF1*/*2a* and other *GRFs* were highly expressed in Pd (Figure 4A, Supplementary Table S7). On the basis of the expression data from different tissues of *P*. *trichocarpa* (https://phytozome.jgi.doe.gov, accessed on 11 November 2020), we compared the expression patterns of the ten *PtrGRF* genes. The results show that *PtrGRF1*/*2a*, *PtrGRF1*/*2b* and *PtrGRF2* were strongly expressed in the early dormant bud, late dormant bud and the young leaf. Additionally, we analyzed the whole gene family of *GRF* in *P. trichocarpa*, *Salix purpurea* and *Arabidopsis thaliana*. A total 40 genes were identified in those species. The phylogenetic tree showed that *PtrGRF12a* and *PtrGRF12b* belong to the same class as *AtGRF9*, while the others were divided into four classes (Figure 4C,D). To further analyze the regulatory relationship between GRF and upstream and downstream genes, a protein–protein interaction (PPI) network was constructed. *PtrGRF1*/*2a*, *PtrGRF1*/*2b* and *PtrGRF8* were co-expressed with *GIF*, while *PtrGRF12a* and *PtrGRF12b* interacted with PHRAGMOPLAST ORIENTING KINESIN 1 (*PAKRPs*), which participated in mitotic cytoskeletal arrays and misplaced cell walls.

### 3.5. Relative Expression of the Differentially Expressed Genes

**Figure 5:**
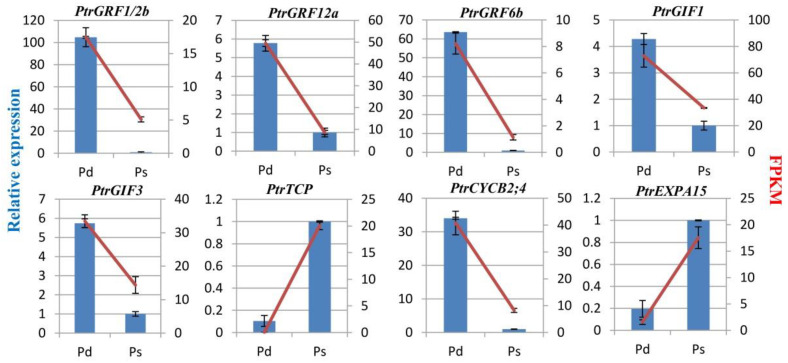
qRT-PCR validation of differentially expressed genes related to leaf size. The left blue histogram represents the qRT-PCR results. The red broken line on the right represents the FPKM of the transcriptome. *PtrGFR1*/*2b* (Potri.014G007200), *PtrGRF6b* (Potri.018G065400), *PtrGRF12a* (Potri.001G114000), *PtrGIF1* (Potri.019G013100), *PtrGIF3* (Potri.002G177600), *PtrTCP* (Potri.T046300), *PtrCYCD2;4* (Potri.002G010000), *PtrEXPA15* (Potri.013G060800). Values are presented as means ± SD of three independent measurements.

To verify the RNA-Seq results, qRT-PCR was used; samples used for qRT-PCR verification were independent of that for RNA-Seq analysis. Three *GRFs*, two *GIFs*, and the cyclin family were selected for qRT-PCR verification (Figure 5). The expression profile of most of the selected genes was similar in the qRT-PCR and RNA-Seq analyses, demonstrating that the RNA-Seq data was reliable.

## 4. Discussion

As an organ of plant photosynthesis, leaf is a nutritive organ with great plasticity in plant evolution, and forms a variety of adaptation types under different selection pressures. Large-leaved species predominate in humid, hot, and sunny environments; small-leaved species are usually distributed in typical hot, dry conditions, and also in high latitudes and elevations [27]. The key indices of drought tolerance in black poplar were the thickness of the leaf cuticle, and the lower and upper palisade, which could be used for the evaluation of drought tolerance of poplar species [28]. In this study, Pd has the typical leaf characteristics of black poplar, such as the upper and lower palisade tissue. Leaf size of Ps was significantly smaller than Pd, but it was thicker (Figure 1 and Table 1). Palisade tissue affects the absorption and utilization of light energy by plants. The thicker palisade tissue is conducive to the absorption of CO_2_, thus promoting the photosynthesis of plants [29,30]. Although the palisade tissue of Ps is thicker and tighter, it only gets the upper layer, while the palisade tissue of Pd and the lower layer contain a large number of chloroplasts (Figure 1B,C). In the early stage of leaves, Pd was red and photosynthetic capacity was weak, which led to the expression of photosynthetic-related genes being lower than that of Ps. Mature leaves are dark green and have palisade tissue of upper and lower beams, which can improve the photosynthetic capacity of mature leaves and thus promote plant growth. The study found that other black poplars also have upper and lower palisade tissues, which may be a special structure of black poplar that will facilitate the rapid growth of plants [28]. At present, studies on leaf cell differentiation mainly focus on guard cell formation, vascular differentiation, and trichome development, etc. [3,31], and the causes for the formation of Pd and other double-layer palisade structures in poplar remain to be further studied.

Phytohormones are important regulators of cell differentiation. Auxin positively regulated S6 kinase and eIF4E-binding proteins (EBP1) and thus increased the rate of translation and ribosome biogenesis [32]. While *ARF2*—a member of a family of transcription factors that mediates gene expression in response to auxin—is a repressor of growth affecting cell division and cell expansion [33,34,35], *WOX5* acts redundantly with *WOX1,* and *WOX3* controls *YUCCA* (*YUC*) auxin biosynthetic gene expression along the leaf margin, thus affecting the leaf shape [36]. In this study, hormone biosynthesis and signal transduction existed in the early stage of leaf development (Supplementary Table S5). Further research is needed on the effect of whole hormone signals on leaf development.

The proper formation of plant tissue is dependent upon a tightly regulated process of cell division. The progression of different phases of the cell cycle requires strict timing regulation of functional proteins involved in DNA replication and mitosis [37]. The increase of nuclear matter content or the change of ploidy often affects the leaf size. For example, triploid poplar can promote the leaf size by affecting the expression of growth hormone, transcription factors and cell differentiation-related genes [38,39]. The complexity of anaphase promoting complex (*APC*) and the cell cycle switch (*CCS52B*) can selectively degrade regulatory protein factors that limit cell division, thus regulating the cell cycle [40,41]. We have identified that the Arabidopsis *PCNA2* homologous gene is highly expressed in Pd, which can form a complex PCNA/CycD with D-cyclin to regulate the cell cycle [42]. Scarecrow (*SCR*), a member of the GRAS family, stimulates S-phase progression of the cell cycle [43]. Additionally, in our study, the DEGs were significantly enriched in ribosome and DNA replication (Figure 3). Therefore, the enhancement of transcription and translation will further enhance gene expression, so as to accelerate cell expansion and increase the number of cells.

TFs, as master regulators, activate or repress a large number of functional genes. In this study, we identified a large number of regulatory genes related to cell differentiation and expansion, including *CYC* and transcription factors (*GRF*, *SCR*, *TCP*) that were significantly differently expressed in Pd and Ps (Figure 4). GRF-GIF regulates the transition between stem cells and their rapidly dividing daughter cells, thus promoting cell proliferation and endowing meristem potential for cell proliferation in organogenesis [44]. *ZmGRF10* increases cell size, while reduces the leaf size and plant height [7]. *OsGRF7* mediates hormone signaling to shorten cell length and control rice architecture by binding the promoter *cytochrome P450* (*OsCYP714B1*) and *OsARF12* [45]. Plant cell wall is an important limiting factor of cell expansion. In cells, the extensibility of the cell wall is dynamically controlled [46]. Expansins play a key role in inducing stress relaxation of the cell wall under expansion pressure, thus limiting cell expansion [47,48]. *PagGRF15* promotes cell expansion by improving the expression of *EXPA11a* and *EXPA11b*, while *PagGRF12a*, *PagGRF12b* positively promote leaf size, mainly through cell proliferation [49,50]. *PagGRF12a* and *PagGRF12b* promote the expression of *CYCB1;1a* and *CYCB1;1b*, while inhibiting *EXP11a* and *EXP11b*, thereby increasing cell number and decreasing cell size in GRF12a and GRF12b overexpression lines [50]. In our results, *PtrGRF12a*, *PtrGRF12b* and *PtrCYC* were highly expressed (Figure 4 and Supplementary Figure S2), and the expression of *EXPA* was the same as that of Wang [50], which suggested that *PtrGRF12a* and *PtrGRF12b* were the principal regulatory genes for leaf size difference between Pd and Ps. Therefore, we infer that there may only be such a regulatory network. *GRFs* were highly expressed in Pd and regulated genes of the cell cycle (like, *CYC*), which accelerates the process of the cell cycle and promotes cell division, but it inhibited the expansion of cells, thus affecting the size of cells, and finally led to the phenotype of small Pd epidermal cells, a large number of cells and a large leaf area.

## 5. Conclusions

In this study, we compared the leaf shape of *P*. *deltoides* ‘Danhong’ and *P*. *simonii* ‘Tongliao1’, and found that the difference in leaf size was caused by the number of cells and cell size. To reveal the molecular mechanism of these phenotypes, RNA-seq was compared between Pd and Ps. Transcriptomic analysis shows genes of ribosomes and the RNA transport pathway were highly expressed in *P*. *deltoides* ‘Danhong’, and identified candidate genes are possibly involved in molecular mechanisms of leaf formation. Furthermore, *PtrGRF1*/*2b*, etc., may function as candidate regulators in cell cycling and expansion in leaf development. These results will promote our understanding of the causes of leaf differences of Pd and Ps, and provide basic data for further research on leaf development regulation.

## Figures and Tables

**Table 1:** Phenotypic character of *P. deltoides* ‘Danhong’ and *P. simonii* ‘Tongliao1’.

Material	Hight (m)	Ground Diameter (mm)	Leaf Length (cm)	Leaf Width (cm)	Leaf Area (cm^2^)	Fresh Weight (g)	Dry Weight (g)	Leaf Mass per Area (mg/cm^2^)	Leaf Thickness (μm)	Thickness of up Palisade Cell (μm)	Thickness of down Palisade Cell (μm)	Cell Size (μm^2^)
Pd	3.47 ± 0.29 **	21.57 ± 2.03 **	19.53 ± 1.48 **	18.45 ± 0.79 **	253.22 ± 33.85 **	5.41 ± 0.88 **	1.70 ± 0.48 **	0.67 ± 0.15	277.00 ± 35.93 **	87.39 ± 7.78 **	72.77 ± 6.99	399.90 ± 35.04 **
Ps	1.55 ± 0.12	9.69 ± 0.89	8.92 ± 0.88	7.24 ± 0.82	45.59 ± 8.08	1.11 ± 0.25	0.32 ± 0.08	0.70 ± 0.09	324.61 ± 40.58	91.74 ± 4.37	0	753.16 ± 82.15

Note: ** indicate *p* < 0.01.

**Table 2:** Summary of DEGs in leaf development.

Gene Id	At	Annotation	log2FC(Pd vs. Ps)	*p*-Value
Potri.013G106800	AT4G16720	ribosomal protein L23/L15e family protein	10.561	2.14 × 10^−203^
Potri.016G076800	AT5G39850	ribosomal protein S4	10.462	3.79 × 10^−120^
Potri.001G360500	AT5G59240	ribosomal protein S8e family protein	9.5602	1.23 × 10^−221^
Potri.006G213300	AT3G55280	ribosomal protein L23AB	5.4155	2.04 × 10^−110^
Potri.017G120200	AT5G58420	ribosomal protein S4 (RPS4A) family protein	4.8474	7.99 × 10^−37^
Potri.015G004700	AT5G24510	60S acidic ribosomal protein family	3.6282	1.68 × 10^−73^
Potri.007G060900	AT4G35830	aconitase 1	−9.7407	0
Potri.006G186800	AT2G24270	aldehyde dehydrogenase 11A3	−8.8477	0
Potri.005G048100	AT3G04940	cysteine synthase D1	−4.1698	8.44 × 10^−33^
Potri.004G054200	AT1G53240	lactate/malate dehydrogenase family protein	−4.0327	9.24 × 10^−49^
Potri.002G007100	AT1G42970	glyceraldehyde-3-phosphate dehydrogenase B subunit	−3.718	2.82 × 10^−35^
Potri.003G088700	AT1G32440	plastidial pyruvate kinase 3	−3.5073	5.87 × 10^−42^

## Data Availability

Data of this project have been deposited with links to SRA accession number (SRR13959619 to SRR13959624) in the National Center for Biotechnology Information (NCBI).

## Supplementary Materials

The following are available online at https://www.mdpi.com/article/10.3390/genes12111775/s1, Figure S1: The photo of *P. deltoides* ‘Danhong’ and *P. simonii* ‘Tongliao1’, Figure S2: The heat map of cyclin dependent kinases in *P. deltoides* ‘Danhong’ and *P. simonii* ‘Tongliao1’, Table S1: Primers used for qRT-PCR experiments, Table S2: Summary of the RNA-seq results, Table S3: The GO enrichement of DEGs between *P. deltoides* ‘Danhong’ and *P. simonii* ‘Tongliao1’, Table S4: The KEGG enrichement of DEGs between *P. deltoides* ‘Danhong’ and *P. simonii* ‘Tongliao1’, Table S5: DEGs of leaf morphological and physiological traits identified by both QTL mapping and transcriptome, Table S6: The expression profiles of the DEGs related to auxin and gibberellins pathways in *P. deltoides* ‘Danhong’ and *P. simonii* ‘Tongliao1’, Table S7: The expression patterns of *GRFs* and cyclins of Pd and Ps in Figure 4 and Figure S2, Table S8: The expression patterns of secondary wall related genes.
