# Supplementary material for: Leaf Size Development Differences and Comparative Transcriptome Analyses of Two Poplar Genotypes
Source: Genes (Basel). 2021 Nov 9;12(11):1775. doi: 10.3390/genes12111775 (PMC8624656; doi:10.3390/genes12111775)
Supplement: Supplementary file 1 [file genes-12-01775-s001.zip › Supplementary Figure S1-2.pdf]

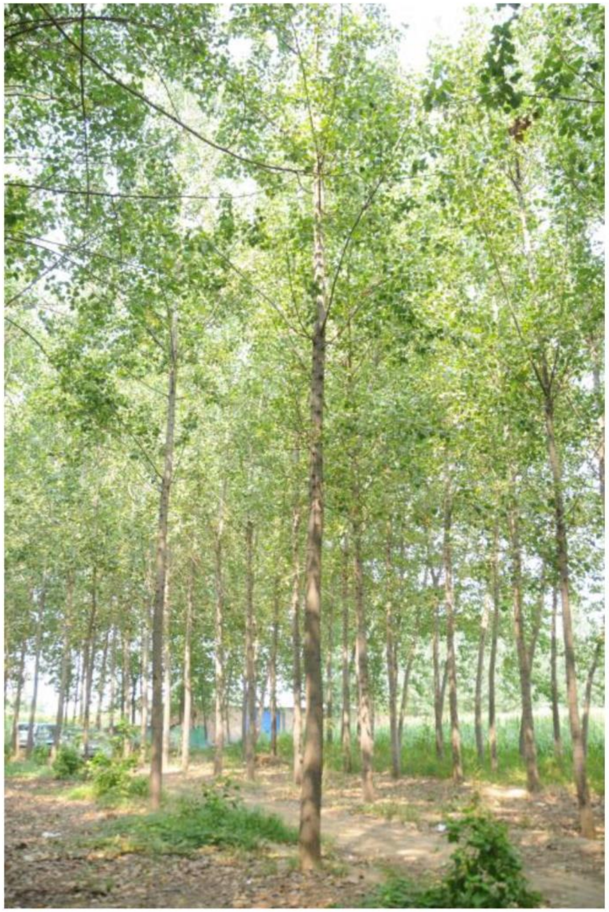

***Populus deltoides* 'Danhong'**

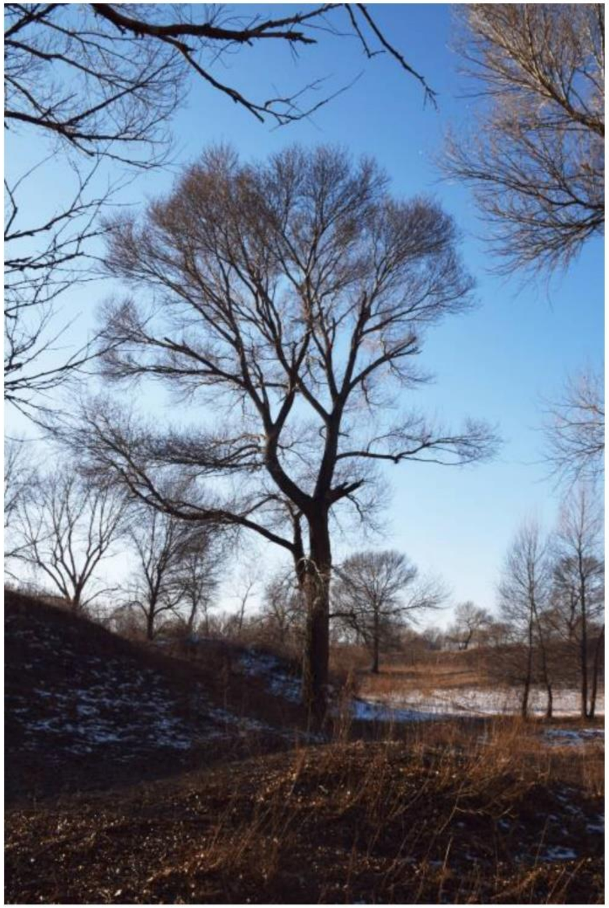

***Populus simonii* 'Tongliao1'**

Figure S1 the photo of *Populus deltoides* 'Danhong' and *Populus simonii* 'Tongliao1'.

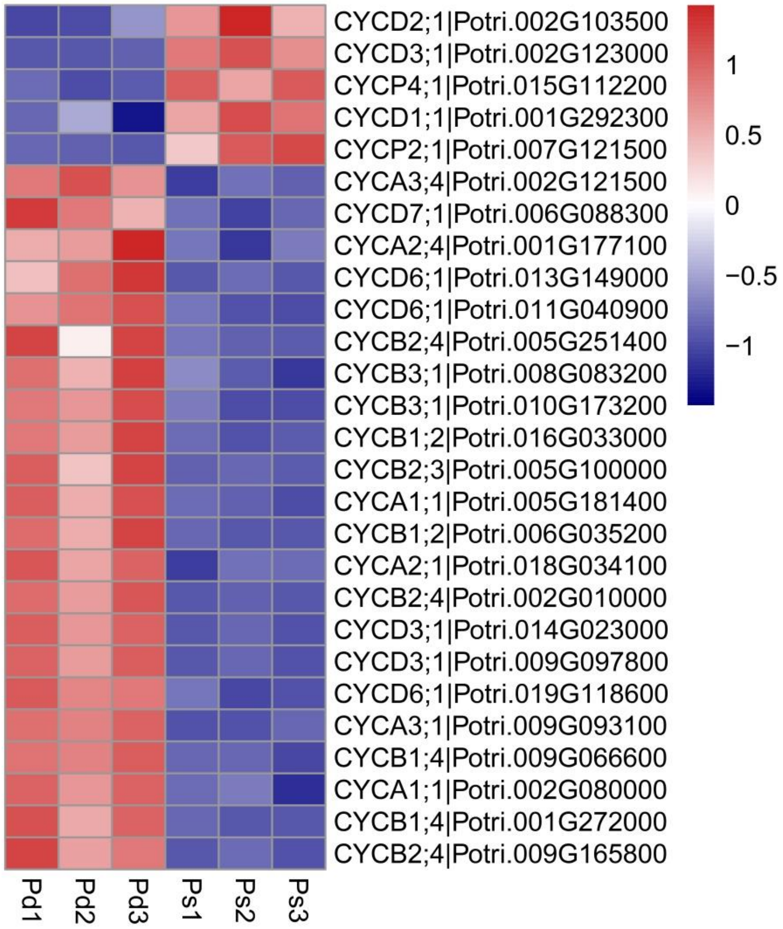

Figure S2 The heat map of cyclin dependent kinases in *P*. *deltoides* ‘Danhong’ and *P. simonii* ‘Tongliao1’.
